# Impact of World Trade Center-Related Health Research: An Application of the NIEHS Translational Framework

**DOI:** 10.3390/ijerph18052659

**Published:** 2021-03-06

**Authors:** Jaime Madrigano, Thomas W. Concannon, Sean Mann, Sameer M. Siddiqi, Ramya Chari, Laura J. Faherty

**Affiliations:** 1 RAND Corporation, Arlington, VA 22202, USA; smann@rand.org (S.M.); ssiddiqi@rand.org (S.M.S.); 2 RAND Corporation, Boston, MA 02116, USA; tconcann@rand.org (T.W.C.); rchari@rand.org (R.C.); lfaherty@rand.org (L.J.F.); 3 Tufts Clinical and Translational Science Institute, Tufts University, Boston, MA 02111, USA; 4 Department of Pediatrics, Boston University School of Medicine, Boston, MA 02118, USA

**Keywords:** World Trade Center, 9/11, disaster, research translation, cancer, PTSD

## Abstract

The World Trade Center Health Program (WTCHP) has a research mission to identify physical and mental health conditions that may be related to the 9/11 terrorist attacks as well as effective diagnostic procedures and treatments for WTC-related health conditions. The ability of the WTCHP to serve its members and realize positive impacts on all of its stakeholders depends on effective translation of research findings. As part of an ongoing assessment of the translational impact of World Trade Center (WTC)-related research, we applied the National Institute of Environmental Health Sciences (NIEHS) translational framework to two case studies: WTC-related research on post-traumatic stress disorder (PTSD) and cancer. We conducted a review of 9/11 health-related research in the peer-reviewed literature through October 2017, grey literature, and WTCHP program documentation. We mapped peer-reviewed studies in the literature to the NIEHS framework and used WTCHP program documentation and grey literature to find evidence of translation of research into clinical practice and policy. Using the NIEHS framework, we identified numerous translational milestones and bridges, as well as areas of opportunity, within each case study. This application demonstrates the utility of the NIEHS framework for documenting progress toward public health impact and for setting future research goals.

## 1. Introduction

The World Trade Center Health Program (WTCHP) provides medical monitoring and treatment for responders at the World Trade Center and related sites and survivors who were in the New York City disaster area. Scientific research supported by the program provides an evidence base for the health monitoring and clinical care of responders and survivors. Through the program’s research mission, physical and mental health conditions that may be related to the 9/11 terrorist attacks are identified and diagnostic procedures and treatments for World Trade Center (WTC)-related health conditions are studied for effectiveness. As part of ongoing work to inform planning activities at the National Institute for Occupational Safety and Health (NIOSH), we are conducting a multi-year, mixed methods study to assess the translational impact of WTC-related health research.

**Figure 1:**
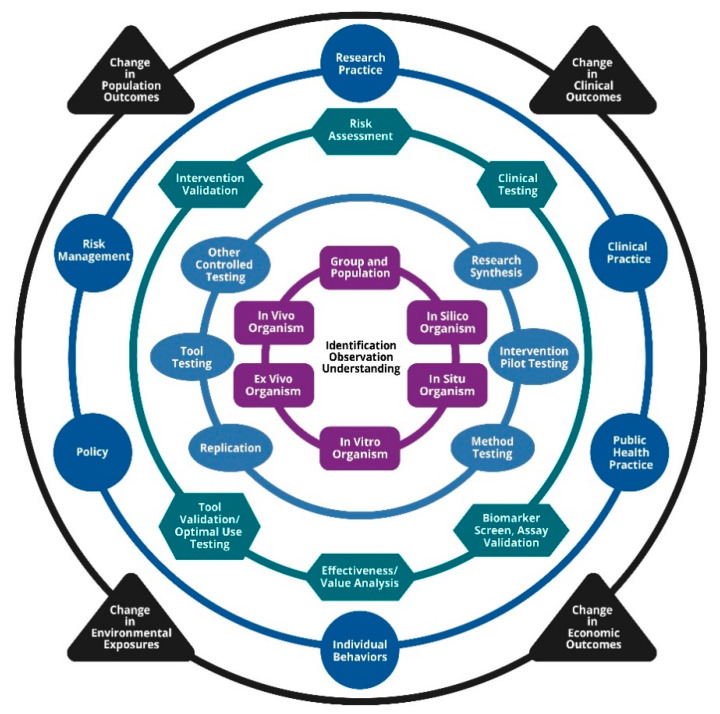
The National Institute of Environmental Health Sciences (NIEHS) Translational Research Framework (reproduced from Environmental Health Perspectives [5]). Environmental Health Perspectives is an open-access journal published with support from the National Institute of Environmental Health Sciences, National Institutes of Health. All content is public domain unless otherwise noted.

Clinical and translational research is the process of turning scientific observations into interventions that improve the health and well-being of individuals and populations [1,2]. To enhance the translation of research into clinical or public health practice, numerous federal agencies have developed programs focused on strengthening translational mechanisms. The National Institute of Health’s National Center for Advancing Translational Sciences (NCATS) developed the National Institutes of Health (NIH) Translational Science Spectrum, which is a commonly used framework that represents research along the path from the biological basis of health and disease to interventions that improve the health of individuals and the public [3]. However, because this framework is centered around clinical applications, it does not adequately represent the paradigm of environmental health research. To more fully address environmental and occupational health research, particularly to capture the complete range of activities within environmental health research at the more basic end of the research spectrum [4], the National Institute of Environmental Health Sciences (NIEHS) published a new framework for translational research in 2018 [5]. This framework is represented by a series of concentric rings that illustrate how ideas and knowledge move from the earliest stages of fundamental questioning to the later stages of impact (Figure 1). Starting from the center and working outward, the series of translational research categories include: Fundamental Questions (purple ring, rectangles), Application and Synthesis (light blue ring, ovals), Implementation and Adjustment (green ring, hexagons), Practice (dark blue ring, circles), and Impact (black ring, triangles). Each ring of the framework includes a series of nodes that identify the types of activities that might occur within each translational research category.

In the NIEHS model, translational research activities and program outcomes within a given research topic area can be mapped to a ring and node on the framework. In describing the use of this framework, NIEHS has noted that research is considered translational when it bridges to another node or translational category within the framework [5]. Thus, we use the concept of translational milestones to define research activities or program outcomes that lead to another research activity or program outcome. The link between translational milestones is defined as a translational bridge. Importantly, the framework does not imply that research should offer complete coverage of every ring and node of the diagram, nor that all research must start at the most basic questions before proceeding to questions on the outer rings. Rather, study of a topic may start on any ring, may skip rings, and may follow a non-linear path.

In our ongoing work conducting a portfolio evaluation of the WTC-related research, we found the NIEHS translational framework to be useful in demonstrating translation, particularly for the early end of the research spectrum (e.g., Fundamental Questions and Application and Synthesis rings), whereas other frameworks were less suitable in capturing these nuances for the breadth of research and related activities contained within this portfolio. We used a literature review in conjunction with the NIEHS translational framework to conduct our assessment, which, to our knowledge, has not previously been done. Given the relative novelty of this framework and its limited application in the peer-reviewed literature to date, it is important to make these findings known to the broader research community. In this paper, we apply the NIEHS translational framework to two topics of particular interest to the WTCHP: research on post-traumatic stress disorder (PTSD) and research on cancer. While these two topics represent only two of many conditions covered by the WTCHP, they offer illustrative examples of research in physical and mental health conditions, topics that are at different stages in their translational trajectory, and were frequently cited as areas of interest by program stakeholders (responders, responders, survivors, clinicians, researchers, and others). We use the NIEHS framework to reveal how WTC-related research has been translated from one type of question or approach to another and to identify future opportunities to increase the translational impact of NIOSH-sponsored research.

## 2. Materials and Methods

### 2.1. Data Sources

This study integrates data from several sources: (1) peer-reviewed, published research funded by NIOSH and other funders on the topics of environmental exposures, health outcomes, health care, and other health-related topics pertaining to WTCHP member populations; (2) non-peer reviewed “grey” literature publications citing or using research to support health-related decisions by stakeholders of the WTCHP; and (3) WTCHP documentation, including on member populations, WTCHP stakeholders, covered conditions, research priorities, and program processes related to research.

### 2.2. Study Methods

Full details of this mixed methods portfolio evaluation will be described in a forthcoming publication upon completion in 2021. However, we have conducted analyses of several streams of data which informed the development of the two case study applications of the NIEHS framework. We briefly describe the data sources and methods of analysis for each of those data streams here. First, we reviewed 9/11 health-related research in the peer-reviewed literature through October 2017 to develop an evidence map of WTC health-related research. An evidence map is a type of systematic review defined as “a systematic search of a broad field to identify gaps in knowledge and/or future research needs that presents results in a user-friendly format, often a visual figure or graph, or a searchable database [6].” In brief, we used a systematic search strategy to find records in databases of peer reviewed publications and grey literature (for details on databases searched and criteria used, see Appendix A and Appendix B). We used five sequential exclusion criteria with increasing specificity to identify studies that were not eligible to be included in this analysis: (1) not in English; (2) not research; (3) not about 9/11 attacks; (4) not about 9/11 populations; (5) not about health conditions, care or outcomes. To guide abstraction of data from peer-reviewed publications, we used a standardized electronic data abstraction form with drop down, numeric, and free text response options. A team of eight reviewers double reviewed included articles and the leadership team (TC, LF, JM) adjudicated reviews. To facilitate comparison across similar studies, we classified publications into three study types. Exposure characterization studies describe physical or chemical stressors in the environment. Exposure-outcome linkage studies examine associations between one or more exposures and health outcomes. Outcome modification studies examine characteristics or impacts of one or more health-related interventions and may include clinical research, health services research, implementation science, or public health or policy interventions. This taxonomy was developed by the research team in order to sufficiently capture the breadth of studies included in this review, although it may limit comparisons to other reviews. For each peer-reviewed publication, we selected one translational ring (fundamental questions, application and synthesis, implementation and adjustment, practice, or impact) and node within the ring that best represented the study’s principal question and approach.

We also analyzed, using a qualitative descriptive approach, the other data sources described above: over 8000 pages of material from grey literature (collected through October 2017) and WTCHP documentation of research activities (collected through January 2020). Briefly, we developed a preliminary codebook, performed an initial round of coding of a randomly selected subset of each category of documents to test and improve the appropriateness of the preliminary codebook, refined the codebook on the basis of these findings, and then completed coding using the revised codebook. We used WTCHP program documentation and grey literature to find evidence of translation of research into clinical practice and policy (e.g., in legislation, covered condition determinations). We used this evidence to describe how and when movement occurred within or between translational rings and nodes of the framework within the case study narratives.

We selected two topics to explore as case studies on research translation: PTSD and cancer. We applied the NIEHS translational research framework in developing these case studies by mapping key translational milestones to the framework’s translational research rings and associated nodes, following steps laid out by NIEHS to guide the use of this framework [7].

## 3. Results

Here, we trace the translational impact of the WTC-related research through two selected topics: research on PTSD and research on cancer.

### 3.1. Translational Story 1: Research on Post-Traumatic Stress Disorder (PTSD)

In the area of WTC-related research on PTSD, the overwhelming majority of research to date has focused on linking 9/11 exposures to the development or prevalence of PTSD in WTC populations. Our preliminary assessment of peer-reviewed literature identified over 200 studies examining PTSD. Of these, 180 (83 percent) were exposure-outcome linkage studies that assessed relationships between WTC-exposures and PTSD as the primary outcome, while only 36 (17 percent) examined clinical, health services, or policy interventions aimed at modifying PTSD.

**Figure 2:**
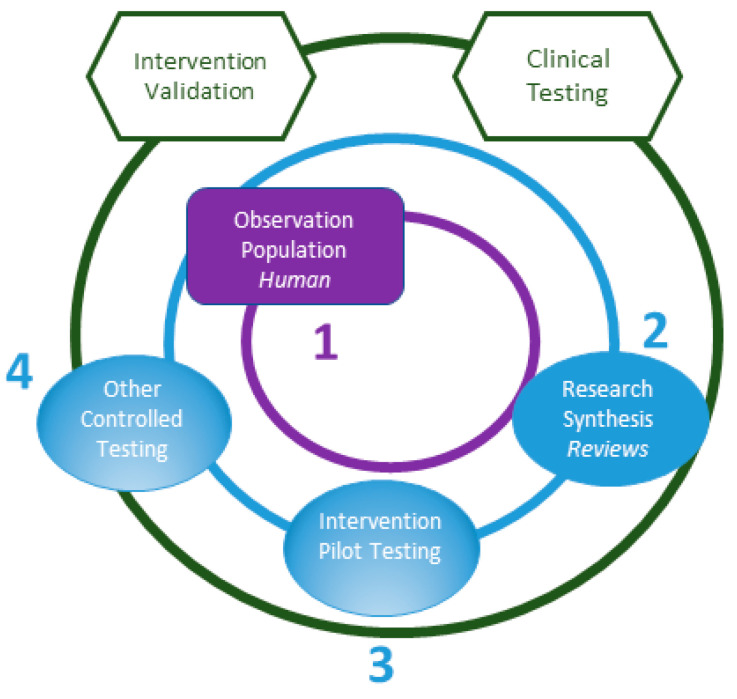
Translational Milestones and Opportunities for Future Translation, Post-Traumatic Stress Disorder (PTSD). Note: shaded nodes represent translational milestones; gradient shaded nodes represent emerging translational milestones; unshaded nodes represent areas of future opportunity for translation.

The vast majority (84%) of exposure-outcome linkage studies on PTSD were population-based observational studies, falling into the Fundamental Questions ring of the NIEHS framework (Figure 2). Much of this research found that PTSD is often one outcome of many following exposure to 9/11. PTSD is co-morbid with and predictive of many medical conditions that have been linked to PTSD in previous work, including respiratory disease, gastroesophageal reflux disease, myocardial infarction, and stroke. This large body of individual exposure-outcome linkage studies motivated a synthesis of the literature, and thus, represents translational milestone 1 (Figure 2).

About 10% of exposure-outcome linkage studies were reviews or syntheses of other studies, which places them into the Application and Synthesis ring of the NIEHS Framework. Thus, for PTSD, we note a translational bridge from individual studies to syntheses of the body of literature relating 9/11 exposures to PTSD. Though not all synthesis studies were high quality systematic reviews, the existence of approximately 20 such studies points to the accumulation over time of a body of evidence relating the 9/11 exposure to the condition of PTSD, which we note as translational milestone 2 (Figure 2).

Of the 36 outcome modification studies addressing PTSD, most were program evaluations or assessments of health services. One in six (17%) examined screening or testing interventions and fewer than one in three (33%) examined clinical interventions. While the clinical interventions cover a range of options—smoking cessation interventions [8], integrative psychotherapy and cognitive behavioral therapy [9,10,11,12,13], virtual reality enhanced exposure therapy [14], pharmacotherapies [15], and a pilot study of a school-based intervention [16]—these studies represent a limited body of literature and suggests that WTC health-related research on PTSD interventions has achieved early milestones but is still emerging. We use gradient shading on the “Intervention Pilot Testing” and “Other Controlled Testing” nodes of the NIEHS Framework to indicate translational milestones 3 and 4, as well as opportunities for future research (Figure 2).

While the body of evidence for PTSD interventions in WTC populations is still in a nascent stage, the WTCHP has made efforts to expand this literature. In January 2012, the WTCHP Scientific Technical Advisory Committee (STAC) had recommended that the WTCHP solicit proposals for “mental health intervention studies.” The March 2012 WTCHP funding opportunity announcement followed by calling for “improvements in diagnosis and treatment” (Cooperative Research Agreements Related to the World Trade Center Health Program {PAR-12-126}). The STAC focused this same recommendation specifically on PTSD in 2014, calling for research on “the effectiveness and utility of PTSD treatments.”

Importantly, findings from recently completed studies funded by the WTCHP and other sources may not yet be published. For instance, three funded interventional studies (or outcome modification studies, in our classification) that examine different therapeutic interventions (mind-body therapies for WTC responders, the Relaxation Response Resiliency Program in Spanish-speaking survivors, and an internet-based cognitive behavioral therapy program for responders) ended between 2017 and 2019.

To continue to advance along the translational spectrum, beyond supporting pilot and controlled testing studies, the WTCHP can explore opportunities for translation around the Implementation and Adjustment ring of the NIEHS Framework. For example, the WTCHP could consider funding studies of PTSD interventions for which current evidence has been supportive in other populations (e.g., veterans) [17]. Other research recommendations supporting “Clinical Testing” studies are documented in the WTCHP Recommendations from Research Meeting Special Sessions [18]. Specifically, the 2018 recommendations from WTC researchers highlight several areas of interest for mental health clinical research, including trials of novel pharmacologic agents and tele-mental health trials. Two nodes on the Implementation and Adjustment ring, “Intervention Validation” and “Clinical Testing,” denoted by unshaded nodes outlined in green in Figure 2, represent prime areas of opportunity for future PTSD-related research within the WTCHP (Figure 2).

### 3.2. Translational Story 2: Research on Cancer

**Figure 3:**
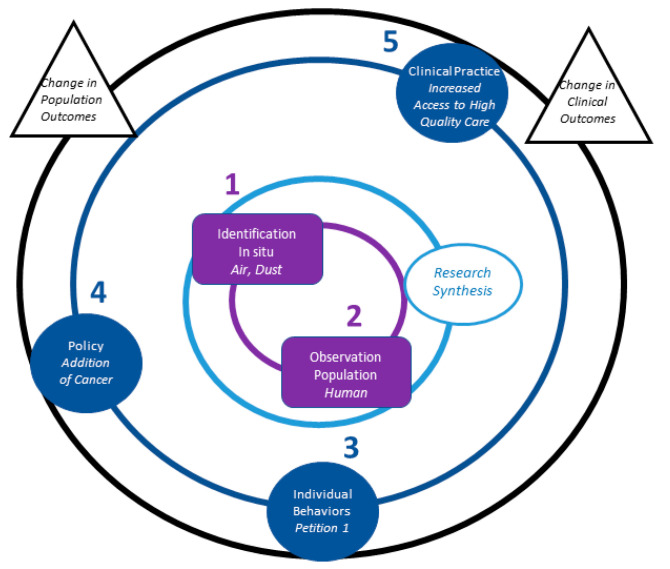
Translational Milestones and Opportunities for Future Translation, Cancer. Note: shaded nodes represent translational milestones; unshaded nodes represent areas of future opportunity for translation.

In July 2011, the WTC Program Administrator released the First Periodic Review of the Scientific and Medical Evidence Related to Cancer for the WTCHP (First Periodic Review), in which available scientific and medical evidence was reviewed to determine if cancer or certain types of cancer should be added to the list of WTC-related health conditions [19]. The First Periodic Review included five peer-reviewed studies of cancer [20,21,22,23,24]: two studies used models to estimate risk of cancer among residents of Lower Manhattan; two were reviews of toxins likely present at Ground Zero, observable short-term health effects, and plausible long-term health effects, including cancer, that might occur; and one was a case series and comparison of rates of multiple myeloma in responders to expected rates in the general population. While these studies provided limited evidence of a link between the WTC terrorist attacks and cancer, most evidence within the First Periodic Review focused on characterizing cancer-related exposures resulting from the WTC attack. These types of studies can be situated in the first ring of the NIEHS framework (Fundamental Questions) because they answer the question, “what is it?” or alternatively, “what is the nature and extent of cancer-related exposures in the WTC terrorist attacks?” We refer to the accumulation of this body of research as translational milestone 1 (Figure 3).

Subsequent to the publication of the First Periodic Review, in September 2011, an epidemiologic study was published by Zeig-Owens et al. [25] that indicated an elevated level of risk of certain types of cancer in responders who served at Ground Zero. The Zeig-Owens et al. publication falls into the category of Observation and answers the question, “what is it doing?” or, alternatively, “what are the outcomes of cancer-related exposures in the WTC terrorist attacks?”, similar to the earlier-published case series of multiple myeloma, but with a more complete description of the population at risk resulting in a more rigorous design. We refer to this publication as translational milestone 2. As translational milestones 1 and 2 cover two types of biomedical research on the Fundamental Questions ring, with the first set of studies determining what exposures existed at the WTC site and the next set of studies following from the first to determine what kind of effect such exposures may have had on exposed populations, there exists a translational bridge between them.

On 7 September 2011, the Administrator of the WTC Health Program received a written petition (Petition 001) to consider adding cancer to the list of WTC-Related Health Conditions, referencing the recently published Zeig-Owens et al. study. The STAC met in November 2011, February 2012, and March 2012 to consider the WTCHP’s request for recommendations on Petition 001. Stakeholders were heavily engaged in this process, some expressing concerns about the length of time between exposure and establishment of this evidence. One stakeholder, a survivor, said, “It’s been over ten years since the World Trade Center was destroyed, and that’s been a time so many first responders have paid with their lives [26].” We refer to the submission of Petition 001 in response to published research (shown as an “individual behavior” node on the Practice ring in Figure 3) as translational milestone 3.

Based, in part, on data from WTC-related scientific publications, including environmental sampling data and epidemiologic studies, and in response to the petition, the WTCHP initially certified 60 types of cancer as WTC-related health conditions and a final rule added them to the list of WTC-related health conditions [27]. Since that time, prostate cancer has been added to the list through subsequent rulemaking. We refer to the certification of these cancers, a policy decision in response to interpretation of available evidence, as translational milestone 4.

To certify these conditions, the WTCHP applied a hazard-based, multiple methods approach, rather than a risk-based approach. A hazard-based approach focuses on identifying whether particular “hazards”—sources of potential harm—are associated with certain health conditions, whereas a risk-based approach would have attempted to quantify the risks of developing those health conditions. The approach relies on four methods, with method 1 being most rigorous and method 4 least rigorous: (1) data from epidemiologic studies supports a causal association between 9/11 exposures and a cancer type; (2) epidemiologic studies support a causal association between a condition already on the list and a cancer type; (3) a hazard is identified at the 9/11 sites that the National Toxicology Program (NTP) identifies as a human carcinogen or has at least limited evidence from the International Agency for Research on Cancer (IARC) that the hazard causes cancer; and (4) the STAC provides a reasonable basis to add the cancer to the list. The vast majority of certified cancers (*n* = 53) were added using Method 3 and none were added using Method 1. We refer to the application of scientific evidence to inform both the submission of Petition 001 and the WTCHP’s certification decisions as additional translational bridges, demonstrating movement from the Fundamental Questions ring outward to the Practice ring. Visualization of these sequential milestones on the NIEHS translational framework in Figure 3 demonstrates how WTC-related research informed decision making.

These translational milestones have had important impacts on clinical care, clinical outcomes, and population outcomes. According to a July 2014 report from the U.S. Government Accountability Office (GAO), adding cancers to the list facilitated the development of cancer-related policies and procedures which, in turn, resulted in access to care, including cancer-related monitoring that could result in early detection of the disease and contribute to better health outcomes for enrollees who otherwise would not have had access to such services [28]. The GAO reported that,

*the addition of cancers to the list has helped to ensure that enrollees have access to high-quality cancer care because providers affiliated with the CCEs have experience treating cancer patients and are well suited for monitoring recurrences and complications… In addition, officials noted that the network established by HealthSmart on behalf of the CCEs includes physicians that specialize in cancer care and provide high-quality services…WTCHP officials and others also reported that the addition of cancers has helped ensure access to high-quality cancer care because the program developed cancer-related policies and procedures using appropriate guidance…*

Thus, we identify a change in clinical practice and care (i.e., increased access to care) as translational milestone 5, and we indicate another translational bridge illustrating movement around the Practice ring of the NIEHS framework (Figure 3).

The existence of IARC’s research documenting known links between environmental exposures and cancer amounted to a rare opportunity to use the principle of biological plausibility to certify cancers as covered conditions. As an internationally recognized authority on links between exposure and cancers, IARC’s work gave WTCHP the solid evidence it needed to proceed with cancer certification. Other conditions will not have the benefit of such an extensive and rigorous review of external research. A representative from the Uniformed Firefighters’ Association noted both this unique opportunity and its life-saving implications during Meeting One of the STAC in 2011,

*This language speaks directly to the intent of Congress to have the basis for inclusion be on biological plausibility… rather than on an exhaustive scientific process which would be completed when few, if any, responders would be alive to avail themselves of the treatment component of the law [29].*

However, this unique opportunity also presented a challenge in that it could set the stage for unrealistic expectations regarding future certification determinations, especially if not communicated clearly. In fact, the above-referenced GAO Report noted that the approach to carve out this exception for cancer certification could have been more clearly communicated to all stakeholders and the lack of clarity could raise questions about the credibility of the approach. NIOSH agreed that future rulemaking would more clearly communicate standards for adding conditions to the list of covered conditions.

In Figure 3, we represent—with unshaded nodes as black triangles on the Impact ring—the Program’s opportunities to change clinical and population outcomes through future research and practice. For instance, the Program could consider funding research to assess cancer monitoring/screening practices or other behavioral interventions for their ability to impact survival and quality of life, as well as funding studies that synthesize evidence of therapeutic interventions for cancer that are relevant to WTC populations.

As visualized through the NIEHS translational framework, it is evident that WTC-related cancer research on fundamental questions has already achieved significant translational milestones, including changes in individual behavior, policy, and clinical practice. There are also emerging opportunities that may be addressed through future research, policy, and planning.

## 4. Discussion

To date there has been limited application of the NIEHS translational research framework [30]. Adding to this literature, we used two case studies to demonstrate how the framework can help identify changes in clinical practice, policy, and individual behaviors that are linked to WTC-related research. We developed these cases studies in conjunction with a literature review, which facilitated the mapping of a large body of literature onto the framework. In addition, using this framework enabled us to highlight potential opportunities to make other translational bridges that will improve clinical and population outcomes. Thus, this framework can be used not only to track progress and achievements in impact that have already been realized, but also to identify new research questions and areas of opportunity for programs such as the WTCHP that are likely to have a significant impact on public health.

In our assessment of two selected topic areas, we found that WTC-related research tended to be concentrated in the Fundamental Questions and Application and Synthesis portions of the NIEHS framework, yet we identified numerous translational bridges within those research categories for both PTSD and cancer. This finding represents another important benefit of using the NIEHS framework that was anticipated in its development. It offers the ability to distinguish and give more recognition to important handoffs and “bridges” that were previously grouped together as “basic” research [7]. While there are opportunities to support further WTC-related research that impacts clinical, population, economic, and environmental outcomes, it is important to recognize the translational milestones that have already been achieved.

Overall, while the NIEHS framework offers a greater level of complexity and nuance than some other frameworks, we found it relatively straightforward to apply to our two case studies. In applying the categories in this framework to the selected studies, our team experienced a high level of agreement regarding which rings studies should be mapped to. In most cases, there was also a high level of agreement on the node within a ring where a study belonged. However, in some cases, particularly for studies which fell within the Practice (dark blue, circles) ring, the line was less clear as to whether a study primarily dealt with public health practice, research practice, or even policy. This is likely primarily due to the complex nature of research itself, which often operates on a continuum, touching on aspects of practice and policy, sometimes in any given study.

In the process of developing these two case studies of translation, we identified several facilitators of and barriers to successful translation of WTCHP-funded research which will be the focus of future work within this ongoing assessment. For example, communication with stakeholders is a key element of research translation and can serve as either a facilitator or barrier, depending on how it is conducted. The inherent limitations of available data from a disaster event, such as 9/11 (both exposure data and that collected from affected individuals), are challenges that can be mitigated but not completely overcome. Importantly, the process of developing a translational story can elucidate these barriers, and thus, can serve as an important process evaluation that may steer a program of research toward the active creation of translational bridges. Furthermore, the NIEHS translational framework can be an important tool in the communication process, providing a unique visual display of how research activities motivate and inform one another, as well as program outcomes. We encourage other researchers to consider using this framework in their communication activities with a wide array of stakeholders, including lay audiences, funders, and other researchers.

## 5. Conclusions

Using the NIEHS framework, we classified WTC-related research in two topic areas according to their translational impact over time. We also identified changes in clinical practice, policy, and individual behaviors that were associated with WTC-related research. Finally, we identified areas of opportunity for future research translation. The application of this framework, as part of a larger mixed methods assessment, proved highly relevant and useful in our effort to categorize where research efforts fall along a translational research spectrum, as other models of translational research fell short in capturing the steps in the translational research process as applied to environmental health. Researchers and research program leaders may wish to consider using the NIEHS framework to communicate the concept of translational research within their program; track movement of research through the translational spectrum; and plan, implement, and evaluate high impact research translation.

## Data Availability

A database of publicly accessible documents used in this research will be available by request upon project completion.

## Appendix A. Search Strategy for Peer-reviewed Publications

*Ovid Medline*

Limit to 2001-present

Statement #1: Common references to the attack

(“world trade center” OR wtc OR (sep* adj1”11”) OR (sep* adj1”11th”) OR “ground zero”).ti,ab,kf. OR September 11 Terrorist Attacks/

Statement #2: Numerical Date and Disaster Terms

NUMERICAL DATE TERMS: “9/11”.ti,ab,kf.

DISASTER TERMS: (terror* OR attack* OR catastroph* OR disaster* OR collaps* OR tower* OR building* OR fire* OR plane* OR airplane* OR jet* OR aircraft* OR burn* OR crash* OR hijack*).ti,ab,kf.

Statement #3: NYC & NJ Location and Disaster Terms

NYC & NJ LOCATION TERMS: ((“new york” OR NY OR NYC OR “new jersey” OR NJ OR “lower Manhattan”).ti,ab,kf. New Jersey/OR New York/)

DISASTER TERMS: (terror* OR attack* OR catastroph* OR disaster* OR crash* OR (building* adj3 collaps*) OR (tower* adj3 collaps*) OR (building* adj3 burn*) OR (tower* adj3 burn*) OR (building* adj3 fire*) OR (tower* adj3 fire*) OR plane* OR airplane* OR jet* OR aircraft* OR “twin tower*” OR hijack*).ti,ab,kf.

Statement #4: Shanksville & Pentagon Locations and Disaster Terms

SHANKSVILLE & PENTAGON LOCATION TERMS: (Shanksville OR “somerset county” OR stonycreek OR pentagon OR “Washington DC” OR “Washington D.C.” OR “district of Columbia” OR Arlington).ti,ab,kf.

DISASTER TERMS: (terror* OR attack* OR catastroph* OR disaster* OR crash* OR (building* adj3 collaps*) OR (tower* adj3 collaps*) OR (building* adj3 burn*) OR (tower* adj3 burn*) OR (building* adj3 fire*) OR (tower* adj3 fire*) OR plane* OR airplane* OR jet* OR aircraft* OR “twin tower*” OR hijack*).ti,ab,kf.

*PsycINFO*

Limit to 2001-present; phrase searching

Statement #1: Common references to the attack

TI “world trade center” OR AB “world trade center” OR KW “world trade center” OR TI “wtc” OR AB “wtc” OR KW “wtc” OR TI(sep* N1 11) OR AB(sep* N1 11) OR KW(sep* N1 11) OR TI(sep* N1 11th) OR AB(sep* N1 11th) OR KW(sep* N1 11th) OR TI “ground zero” OR AB “ground zero” OR KW “ground zero”

Statement #2: Numerical Date and Disaster Terms

NUMERICAL DATE TERMS: TI 9/11 OR AB 9/11 OR KW 9/11

DISASTER TERMS: TI terror* OR AB terror* KW terror* OR TI attack* OR AB attack* OR KW attack* OR TI catastroph* OR AB catastroph* OR KW catastroph* OR TI disaster* OR AB disaster* OR KW disaster* OR TI collapse* OR AB collapse* OR KW collapse* OR TI tower* OR AB tower* OR KW tower* OR TI building* OR AB building* OR KW building* OR TI fire* OR AB fire* OR KW fire* OR TI plane* OR AB plane* OR KW plane* OR TI airplane* OR KW airplane* OR TI jet* OR AB jet* OR KW jet* OR TI aircraft* OR AB aircraft* OR aircraft* OR TI burn* OR AB burn* OR KW burn* OR TI crash* OR AB crash* OR KW crash* OR TI hijack* OR AB hijack* OR KW hijack*

Statement #3: NYC & NJ Location and Disaster Terms

NYC & NJ LOCATION TERMS: TI “New York” OR AB “New York” OR KW “New York” OR TI NY OR AB NY OR KW NY OR TI “New Jersey” OR AB “New Jersey” OR KW “New Jersey” OR TI NJ OR AB NJ OR KW NJ OR TI “lower manhattan” OR AB “Lower manhattan” OR KW “Lower Manhattan”

DISASTER TERMS: TI terror* OR AB terror* KW terror* OR TI attack* OR AB attack* OR KW attack* OR TI catastroph* OR AB catastroph* OR KW catastroph* OR TI disaster* OR AB disaster* OR KW disaster* OR TI collapse* OR AB collapse* OR KW collapse* OR TI tower* OR AB tower* OR KW tower* OR TI building* OR AB building* OR KW building* OR TI fire* OR AB fire* OR KW fire* OR TI plane* OR AB plane* OR KW plane* OR TI airplane* OR KW airplane* OR TI jet* OR AB jet* OR KW jet* OR TI aircraft* OR AB aircraft* OR aircraft* OR TI burn* OR AB burn* OR KW burn* OR TI crash* OR AB crash* OR KW crash* OR TI hijack* OR AB hijack* OR KW hijack*

Statement #4: Shanksville & Pentagon Locations and Disaster Terms

SHANKSVILLE & PENTAGON LOCATION TERMS: TI Shanksville OR AB Shanksville OR KW Shanksville OR TI Stonycreek OR AB stonycreek OR KW Stonycreek OR TI pentagon OR AB pentagon OR KW pentagon OR TI “Washington DC” OR AB “Washington DC” OR KW “Washington DC” OR TI “Washington D.C.” OR AB “Washington D.C.” OR KW “Washington D.C.” OR TI “District of Columbia” OR AB “District of Columbia” OR KW “District of Columbia” OR TI Arlington OR AB Arlington OR KW Arlington

DISASTER TERMS: TI terror* OR AB terror* OR KW terror* OR TI attack* OR AB attack* OR KW attack* OR TI catastroph* OR AB catastroph* OR KW catastroph* OR TI disaster* OR AB disaster* OR KW disaster* OR TI crash* OR AB crash* OR TI crash* OR TI(building* N3 collaps*) OR AB(building* N3 collaps*) OR KW(building* N3 collaps*) OR TI(tower* N3 collaps*) OR AB(tower* N3 collaps*) OR KW (tower* N3 collaps*) OR TI(building* N3 burn*) OR AB(building* N3 burn*) OR KW(building* N3 burn*)OR TI(tower* N3 burn*) OR AB(tower* N3 burn*) OR KW(tower* N3 burn*) OR TI(building* N3 fire*) OR AB(building N3 fire*) OR KW(building N3 fire*) OR TI(tower* N3 fire*) OR AB(tower* N3 fire*) OR KW(tower* N3 fire*)OR TI plane* OR AB plane* OR KW plane* OR TI airplane* OR KW airplane* OR AB airplane* OR TI jet* OR AB jet* OR KW jet* OR TI aircraft* OR AB aircraft* OR KW aircraft* OR AB “twin tower” OR AB “twin tower” OR KW “twin tower” OR TI hijack* OR AB hijack* OR KW hijack*

*CINAHL*

Limit to 2001-present; phrase searching; exclude medline records

Statement #1: Common references to the attack

TI “world trade center” OR AB “world trade center” OR MW “world trade center” OR TI “wtc” OR AB “wtc” OR MW “world trade center” OR TI(sep* N1 11) OR AB(sep* N1 11) OR MW(sep* N1 11) OR TI(sep* N1 11th) OR AB(sep* N1 11th) OR MW(sep* N1 11th) OR TI “ground zero” OR AB “ground zero” OR MW “ground zero”

Statement #2: Numerical Date and Disaster Terms

NUMERICAL DATE TERMS: TI 9/11 OR AB 9/11 OR MW 9/11

DISASTER TERMS: TI terror* OR AB terror* OR MW terror* OR TI attack* OR AB attack* OR MW attack* OR TI catastroph* OR AB catastroph* OR MW catastroph* OR TI disaster* OR AB disaster* OR MW disaster OR TI collaps* OR AB collaps* OR MW collaps* OR TI tower* OR AB tower* OR MW tower& OR TI building* OR AB building* OR MW building* OR TI fire* OR AB fire* OR MW fire* OR TI plane* OR AB plane* OR MW plane* OR TI airplane* OR AB airplane* OR MW airplane* OR TI jet* OR AB jet* OR MW jet* OR TI aircraft* OR AB aircraft* OR MW aircraft* OR TI burn* OR AB burn* OR MW burn* OR TI crash* OR AB crash* OR MW crash* OR TI hijack* OR AB hijack* OR MW hijack*

Statement #3: NYC & NJ Location and Disaster Terms

NYC & NJ LOCATION TERMS: TI “New York” OR AB “New York” MW “new York” OR TI NY OR AB NY OR MW NY OR TI “New Jersey” OR AB “New Jersey” OR MW “new jersey” OR TI NJ OR AB NJ MW NJ OR TI “lower Manhattan” OR AB “Lower Manhattan” OR KW “Lower Manhattan”

DISASTER TERMS: TI terror* OR AB terror* OR MW terror* OR TI attack* OR AB attack* OR MW attack* OR TI catastroph* OR AB catastroph* OR MW catastroph* OR TI disaster* OR AB disaster* OR MW disaster* OR TI crash* OR AB crash* OR MW crash* OR TI(building* N3 collaps*) OR AB(building* N3 collaps*) OR MW(building* N3 collaps*) OR TI(tower* N3 collaps*) OR AB(tower* N3 collaps*) OR MW(tower* N3 collaps*) OR TI(building* N3 burn*) OR AB(building* N3 burn*) OR MW(building* N3 burn*) OR TI(tower* N3 burn*) OR AB(tower* N3 burn*) OR MW(tower* N3 burn*) OR TI(building* N3 fire*) OR AB(building N3 fire*) OR MW(building N3 fire*) OR TI(tower* N3 fire*) OR AB(tower* N3 fire*) OR MW(tower* N3 fire*) OR TI plane* OR AB plane* OR MW plane* OR TI airplane* OR AB airplane* OR MW airplane* OR TI jet* OR AB jet* OR MW jet* OR TI aircraft* OR AB aircraft* OR MW aircraft* OR AB “twin tower” OR AB “twin tower” OR MW “twin tower” OR TI hijack* OR AB hijack* OR MW hijack*

Statement #4: Shanksville & Pentagon Locations and Disaster Terms

SHANKSVILLE & PENTAGON LOCATION TERMS: TI Shanksville OR AB Shanksville OR MW Shanksville OR TI Stonycreek OR AB stonycreek OR MW Shanksville OR TI pentagon OR AB pentagon OR MW pentagon OR TI “Washington DC” OR AB “Washington DC” OR MW “Washington DC” OR TI “Washington D.C.” OR AB “Washington D.C.” OR MW “Washington D.C.” OR TI “District of Columbia” OR AB “District of Columbia” OR MW “District of Columbia” OR TI Arlington OR AB Arlington OR MW Arlington

DISASTER TERMS: TI terror* OR AB terror* OR MW terror* OR TI attack* OR AB attack* OR MW attack* OR TI catastroph* OR AB catastroph* OR MW catastroph* OR TI disaster* OR AB disaster* OR MW disaster* OR TI crash* OR AB crash* OR MW crash* OR TI(building* N3 collaps*) OR AB(building* N3 collaps*) OR MW(building* N3 collaps*) OR TI(tower* N3 collaps*) OR AB(tower* N3 collaps*) OR MW(tower* N3 collaps*) OR TI(building* N3 burn*) OR AB(building* N3 burn*) OR MW(building* N3 burn*) OR TI(tower* N3 burn*) OR AB(tower* N3 burn*) OR MW(tower* N3 burn*) OR TI(building* N3 fire*) OR AB(building N3 fire*) OR MW(building N3 fire*) OR TI(tower* N3 fire*) OR AB(tower* N3 fire*) OR MW(tower* N3 fire*) OR TI plane* OR AB plane* OR MW plane* OR TI airplane* OR AB airplane* OR MW airplane* OR TI jet* OR AB jet* OR MW jet* OR TI aircraft* OR AB aircraft* OR MW aircraft* OR AB “twin tower” OR AB “twin tower” OR MW “twin tower” OR TI hijack* OR AB hijack* OR MW hijack*

*Scopus*

Limit 2001-present

(notes: remove WTC from statement #1, change sep* to sept*)

Statement #1: Common references to the attack

TITLE-ABS-KEY(“world trade center”) OR TITLE-ABS-KEY (sept* W/0 11) OR TITLE-ABS-KEY (“ground zero”) AND (EXCLUDE (SUBJAREA, “SOCI”) OR EXCLUDE (SUBJAREA, “ENGI”) OR EXCLUDE (SUBJAREA, “ARTS”) OR EXCLUDE (SUBJAREA, “COMP”) OR EXCLUDE (SUBJAREA, “EART”) OR EXCLUDE (SUBJAREA, “MATE”) OR EXCLUDE (SUBJAREA, “PHYS”) OR EXCLUDE (SUBJAREA, “AGRI”) OR EXCLUDE (SUBJAREA, “CENG”) OR EXCLUDE (SUBJAREA, “ENER”) OR EXCLUDE (SUBJAREA, “MATH”) OR EXCLUDE (SUBJAREA, “CHEM”) OR EXCLUDE (SUBJAREA, “VETE”) OR EXCLUDE (SUBJAREA, “Undefined”)) AND (EXCLUDE (SRCTYPE, “Undefined”))

Statement #2: Numerical Date and Disaster Terms

NUMERICAL DATE TERM: TITLE-ABS-KEY (“9/11”)

DISASTER TERMS: TITLE-ABS-KEY (terror* OR attack* OR catastroph* OR disaster* OR collaps* OR tower* OR building* OR fire* OR plane* OR airplane* OR jet* OR aircraft* OR burn* OR crash* OR hijack*) OR ABS(terror* OR attack* OR catastroph* OR disaster* OR collaps* OR tower* OR building* OR fire* OR plane* OR airplane* OR jet* OR aircraft* OR burn* OR crash* OR hijack*) AND (EXCLUDE (SUBJAREA, “SOCI”) OR EXCLUDE (SUBJAREA, “ARTS”) OR EXCLUDE (SUBJAREA, “COMP”) OR EXCLUDE (SUBJAREA, “EART”) OR EXCLUDE (SUBJAREA, “PHYS”) OR EXCLUDE (SUBJAREA, “CHEM”) OR EXCLUDE (SUBJAREA, “MATE”) OR EXCLUDE (SUBJAREA, “CENG”) OR EXCLUDE (SUBJAREA, “ENER”) OR EXCLUDE (SUBJAREA, “AGRI”) OR EXCLUDE (SUBJAREA, “MATH”) OR EXCLUDE (SUBJAREA, “VETE”))

Statement #3: NYC & NJ Location and Disaster Terms

NYC & NJ LOCATION TERMS: TITLE-ABS-KEY(“new york” OR NY OR NYC OR “new jersey” OR NJ OR “lower Manhattan”) AND (EXCLUDE (SUBJAREA, “ENGI”) OR EXCLUDE (SUBJAREA, “COMP”) OR EXCLUDE (SUBJAREA, “PHYS”) OR EXCLUDE (SUBJAREA, “SOCI”) OR EXCLUDE (SUBJAREA, “MATE”) OR EXCLUDE (SUBJAREA, “MATH”) OR EXCLUDE (SUBJAREA, “ARTS”) OR EXCLUDE (SUBJAREA, “CHEM”) OR EXCLUDE (SUBJAREA, “EART”) OR EXCLUDE (SUBJAREA, “AGRI”) OR EXCLUDE (SUBJAREA, “CENG”) OR EXCLUDE (SUBJAREA, “ENER”) OR EXCLUDE (SUBJAREA, “VETE”) OR EXCLUDE (SUBJAREA, “Undefined”)) AND (EXCLUDE (SRCTYPE, “Undefined”))

DISASTER TERMS: TITLE-ABS-KEY (terror* OR attack* OR catastroph* OR disaster* OR crash* OR plane* OR airplane* OR jet* OR aircraft* OR “twin tower*” OR hijack*) OR TITLE-ABS-KEY (building w/3 collaps*) OR TITLE-ABS-KEY(tower* w/3 collaps*) OR TITLE-ABS-KEY(building* w/3 burn*) OR TITLE-ABS-KEY(tower* w/3 burn*) OR TITLE-ABS-KEY(building* w/3 fire*) OR TITLE-ABS-KEY(tower* w/3 fire*)

Statement #4: Shanksville & Pentagon Locations and Disaster Terms

SHANKSVILLE & PENTAGON LOCATION TERMS: TITLE-ABS-KEY (Shanksville OR “somerset county” OR stonycreek OR pentagon OR “Washington DC” OR “Washington D.C.” OR “district of Columbia” OR Arlington) AND (EXCLUDE (SUBJAREA, “ENGI”) OR EXCLUDE (SUBJAREA, “SOCI”) OR EXCLUDE (SUBJAREA, “PHYS”) OR EXCLUDE (SUBJAREA, “ARTS”) OR EXCLUDE (SUBJAREA, “COMP”) OR EXCLUDE (SUBJAREA, “MATH”) OR EXCLUDE (SUBJAREA, “EART”) OR EXCLUDE (SUBJAREA, “MATE”) OR EXCLUDE (SUBJAREA, “CHEM”) OR EXCLUDE (SUBJAREA, “CENG”) OR EXCLUDE (SUBJAREA, “ENER”) OR EXCLUDE (SUBJAREA, “AGRI”) OR EXCLUDE (SUBJAREA, “VETE”) OR EXCLUDE (SUBJAREA, “Undefined”)) AND (LIMIT-TO (DOCTYPE, “cp”))

DISASTER TERMS: TITLE-ABS-KEY (terror* OR attack* OR catastroph* OR disaster* OR crash* OR plane* OR airplane* OR jet* OR aircraft* OR “twin tower*” OR hijack*) OR TITLE-ABS-KEY (building w/3 collaps*) OR TITLE-ABS-KEY(tower* w/3 collaps*) OR TITLE-ABS-KEY(building* w/3 burn*) OR TITLE-ABS-KEY(tower* w/3 burn*) OR TITLE-ABS-KEY(building* w/3 fire*) OR TITLE-ABS-KEY(tower* w/3 fire*)

*Web of Science*

Limit to 2001-present

Indexes = SCI-EXPANDED, CPCI-SSH, BKCI-S, IC, Timespan = 2001–2017

(notes: remove WTC from statement #1, changed Sep* to Sept*.)

Statement #1: Common references to the attack

TS = (“world trade center”) OR TS = (sept* NEAR/0 11) OR TS = (sept* NEAR/0 11th) OR TS = (“ground zero”) Exclude: RESEARCH AREAS: (PLANT SCIENCES OR ENGINEERING OR REMOTE SENSING OR COMPUTER SCIENCE OR ZOOLOGY OR MARINE FRESHWATER BIOLOGY OR HISTORY PHILOSOPHY OF SCIENCE OR ASTRONOMY ASTROPHYSICS OR EDUCATION EDUCATIONAL RESEARCH OR AGRICULTURE OR MATERIALS SCIENCE OR PHYSICAL GEOGRAPHY OR ENERGY FUELS OR FOOD SCIENCE TECHNOLOGY OR CONSTRUCTION BUILDING TECHNOLOGY OR SCIENCE TECHNOLOGY OTHER TOPICS OR MATHEMATICAL METHODS IN SOCIAL SCIENCES OR NUCLEAR SCIENCE TECHNOLOGY OR INFORMATION SCIENCE LIBRARY SCIENCE OR CHEMISTRY OR GEOCHEMISTRY GEOPHYSICS OR THERMODYNAMICS OR PHYSICS OR OPTICS OR METEOROLOGY ATMOSPHERIC SCIENCES OR OCEANOGRAPHY OR ROBOTICS OR MATHEMATICS OR VETERINARY SCIENCES OR MECHANICS OR TELECOMMUNICATIONS OR AUTOMATION CONTROL SYSTEMS OR SPECTROSCOPY OR GEOLOGY OR FISHERIES OR WATER RESOURCES)

Statement #2: Numerical Date and Disaster Terms

NUMERICAL DATE TERMS: TS = (“9/11”)

DISASTER TERMS: TS = (terror* OR attack* OR catastrophe* OR disaster* OR collaps* OR tower* OR building* OR fire* OR plane* OR airplane* OR jet* OR aircraft* OR burn* OR crash* OR hijack*)

Statement #3: NYC & NJ Location and Disaster Terms

NYC & NJ LOCATION TERMS: TS = (“new york” OR NY OR NYC OR “new jersey” OR NJ OR “lower Manhattan”) [excluding] RESEARCH AREAS: (ACOUSTICS OR ENGINEERING OR MATERIALS SCIENCE OR ENVIRONMENTAL SCIENCES ECOLOGY OR OPTICS OR METEOROLOGY ATMOSPHERIC SCIENCES OR GEOLOGY OR VETERINARY SCIENCES OR AGRICULTURE OR MATHEMATICS OR MARINE FRESHWATER BIOLOGY OR PLANT SCIENCES OR INSTRUMENTS INSTRUMENTATION OR PHYSICS OR REMOTE SENSING OR FORESTRY OR HISTORY PHILOSOPHY OF SCIENCE OR ENERGY FUELS OR THERMODYNAMICS OR ENTOMOLOGY OR WATER RESOURCES OR ASTRONOMY ASTROPHYSICS OR AUTOMATION CONTROL SYSTEMS OR ZOOLOGY OR OCEANOGRAPHY OR CRYSTALLOGRAPHY OR CHEMISTRY OR GEOCHEMISTRY GEOPHYSICS OR ANTHROPOLOGY OR CONSTRUCTION BUILDING TECHNOLOGY OR PHYSICAL GEOGRAPHY)

DISASTER TERMS: TS = (terror* OR attack* OR catastroph* OR disaster* OR crash*) OR TS = (building* NEAR/3 collaps*) OR TS = (tower* NEAR/3 collaps*) OR TS = (building* NEAR/3 burn*) OR TS = (tower* NEAR/3 burn*) OR TS = (building* NEAR/3 fire*) OR TS = (tower* NEAR/3 fire*) OR TS = (plane* OR airplane* OR jet* OR aircraft* OR “twin tower*” OR hijack*)

Statement #4: Shanksville & Pentagon Locations and Disaster Terms

SHANKSVILLE & PENTAGON LOCATION TERMS: TS = (Shanksville OR “somerset county” OR stonycreek OR pentagon OR “Washington DC” OR “Washington D.C.” OR “district of Columbia” OR Arlington) [excluding] RESEARCH AREAS: (ACOUSTICS OR ENGINEERING OR MATERIALS SCIENCE OR ENVIRONMENTAL SCIENCES ECOLOGY OR OPTICS OR METEOROLOGY ATMOSPHERIC SCIENCES OR GEOLOGY OR VETERINARY SCIENCES OR AGRICULTURE OR MATHEMATICS OR MARINE FRESHWATER BIOLOGY OR PLANT SCIENCES OR INSTRUMENTS INSTRUMENTATION OR PHYSICS OR REMOTE SENSING OR FORESTRY OR HISTORY PHILOSOPHY OF SCIENCE OR ENERGY FUELS OR THERMODYNAMICS OR ENTOMOLOGY OR WATER RESOURCES OR ASTRONOMY ASTROPHYSICS OR AUTOMATION CONTROL SYSTEMS OR ZOOLOGY OR OCEANOGRAPHY OR CRYSTALLOGRAPHY OR CHEMISTRY OR GEOCHEMISTRY GEOPHYSICS OR ANTHROPOLOGY OR CONSTRUCTION BUILDING TECHNOLOGY OR PHYSICAL GEOGRAPHY)

DISASTER TERMS: TS = (terror* OR attack* OR catastroph* OR disaster* OR crash*) OR TS = (building* NEAR/3 collaps*) OR TS = (tower* NEAR/3 collaps*) OR TS = (building* NEAR/3 burn*) OR TS = (tower* NEAR/3 burn*) OR TS = (building* NEAR/3 fire*) OR TS = (tower* NEAR/3 fire*) OR TS = (plane* OR airplane* OR jet* OR aircraft* OR “twin tower*” OR hijack*)

*Embase*

limit 2001-present; humans

Statement #1: Common references to the attack

(“world trade center” OR (sept* near/1 “11”) OR (sept* near/1”11th”) OR “ground zero”):ti,ab,kw

Statement #2: Numerical Date and Disaster Terms

NUMERICAL DATE TERM: (“9/11”):ti,ab,kw

DISASTER TERMS: (terror* OR attack* OR catastroph* OR disaster* OR collaps* OR tower* OR building* OR fire* OR plane* OR airplane* OR jet* OR aircraft* OR burn* OR crash* OR hijack*):ti,ab,kw

Statement #3: NYC & NJ Location and Disaster Terms

NYC & NJ LOCATION TERMS: (“new york” OR NY OR NYC OR “new jersey” OR NJ OR “lower Manhattan”):ti,ab,kw

DISASTER TERMS: (terror* OR attack* OR catastroph* OR disaster* OR crash* OR (building* adj3 collaps*) OR (tower* adj3 collaps*) OR (building* near/3 burn*) OR (tower* near/3 burn*) OR (building* near/3 fire*) OR (tower* near/3 fire*) OR plane* OR airplane* OR jet* OR aircraft* OR “twin tower*” OR hijack*):ti,ab,kw

Statement #4: Shanksville & Pentagon Locations and Disaster Terms

SHANKSVILLE & PENTAGON LOCATION TERMS: (Shanksville OR “somerset county” OR stonycreek OR pentagon OR “Washington DC” OR “Washington D.C.” OR “district of Columbia” OR Arlington):ti,ab,kw

DISASTER TERMS: (terror* OR attack* OR catastroph* OR disaster* OR crash* OR (building* near/3 collaps*) OR (tower* near/3 collaps*) OR (building* near/3 burn*) OR (tower* near/3 burn*) OR (building* near/3 fire*) OR (tower* near/3 fire*) OR plane* OR airplane* OR jet* OR aircraft* OR “twin tower*” OR hijack*):ti,ab,kw

## Appendix B. Search Strategy for Grey Literature

*Google*

Limit 2001-present

Attack Terms

“World Trade Center” OR WTC OR “September 11” OR “ground zero”

Effect Terms

health; exposure; monitoring; health care; risk

Exposure Terms

chemical; asbestos; dust; particle

Response Terms

prepared; preparedness; response; recovery

Population Terms

worker; responder; survivor

Translation Terms

research translation; research impact; research transparency; research communication; research to clinical practice; research relevance; research intervention; research to practice

Searches took the following form:

(“World Trade Center” OR WTC OR “September 11” OR “ground zero”) AND Effect Term*

(“World Trade Center” OR WTC OR “September 11” OR “ground zero”) AND Exposure Term*

(“World Trade Center” OR WTC OR “September 11” OR “ground zero”) AND Response Term*

(“World Trade Center” OR WTC OR “September 11” OR “ground zero”) AND Population Term*

(“World Trade Center” OR WTC OR “September 11” OR “ground zero”) AND Translation Term*

*Terms were searched one at a time

For each search, three separate search conditions were applied: (1) limiting search results to org; (2) limiting search results to gov; and (3) no limits.

*Homeland Security Digital Library (HSDL)*

Limit 2001-present

Attack Terms

“World Trade Center” OR WTC OR “September 11” OR “ground zero”

Effect Terms

health; exposure; monitoring; health care; risk

Exposure Terms

chemical; asbestos; dust; particle

Translation Terms

research translation; research impact; research transparency; research communication; research to clinical practice; research relevance; research intervention; research to practice

Searches took the following form:

(“World Trade Center” OR WTC OR “September 11” OR “ground zero”) AND Effect Term*

(“World Trade Center” OR WTC OR “September 11” OR “ground zero”) AND Exposure Term*

(“World Trade Center” OR WTC OR “September 11” OR “ground zero”) AND Translation Term*

*Terms were searched one at a time

*govinfo*

Limit 2001-present

Attack Terms

“World Trade Center” OR WTC OR “September 11” OR “ground zero”

Effect Terms

health; exposure; monitoring; health care; risk

Exposure Terms

chemical; asbestos; dust; particle

Searches took the following form:

(“World Trade Center” OR WTC OR “September 11” OR “ground zero”) AND (Effect Terms OR Exposure Terms)

*Harvard Kennedy School Think Tank Search*

Limit 2001-present

Attack Terms

“World Trade Center” OR WTC OR “September 11” OR “ground zero”

Effect Terms

health; exposure; monitoring; health care; risk

Exposure Terms

chemical; asbestos; dust; particle

Response Terms

prepared; preparedness; response; recovery

Population Terms

worker; responder; survivor

Translation Terms

research translation; research impact; research transparency; research communication; research to clinical practice; research relevance; research intervention; research to practice

Searches took the following form:

(“World Trade Center” OR WTC OR “September 11” OR “ground zero”) AND Effect Term*

(“World Trade Center” OR WTC OR “September 11” OR “ground zero”) AND Exposure Term*

(“World Trade Center” OR WTC OR “September 11” OR “ground zero”) AND Response Term*

(“World Trade Center” OR WTC OR “September 11” OR “ground zero”) AND Population Term*

(“World Trade Center” OR WTC OR “September 11” OR “ground zero”) AND Translation Term*

*Terms were searched one at a time
